# Starch as the Flame Retardant for Electrolytes in Lithium-Ion Cells

**DOI:** 10.3390/ma15020523

**Published:** 2022-01-10

**Authors:** Marita Pigłowska, Beata Kurc, Łukasz Rymaniak

**Affiliations:** 1 Institute of Chemistry and Electrochemistry, Faculty of Chemical Technology, Poznan University of Technology, Berdychowo 4, PL-60965 Poznan, Poland; marita.piglowska@student.put.poznan.pl; 2 Institute of Combustion Engines and Powertrains, Faculty of Civil and Transport Engineering, Poznan University of Technology, Piotrowo 3, PL-60965 Poznan, Poland; lukasz.rymaniak@put.poznan.pl

**Keywords:** starch, EIS, flame retardant, viscosity, flash point, electrolyte, lithium-ion battery

## Abstract

The main purpose of this work is to illustrate the flame retardant properties of corn starch that is used as an additive to the classic electrolytes in lithium-ion cells. The advantages of using natural biomass include the increased biodegradability of the cell, compliance with the slogan of green chemistry, as well as the widespread availability and easy isolation of this ingredient. Due to the non-Newtonian properties of starch, it increases work safety and prevents the occurrence of thermal runaway as a shear-thinning fluid in the event of a collision. Thus, its use may, in the future, prevent explosions that affect electric cars with lithium-ion batteries without significantly degrading the electrochemical parameters of the cell. In the manuscript, the viscosity test, flash point measurements, the SET (self-extinguishing time) test and conductivity measurements were performed, in addition to the determination of electrochemical impedance spectroscopy (EIS) for the anode system. Additionally, the kinetic and thermodynamic parameters, for both flow and conductivity, were determined for a deeper analysis; this constitutes the scientific novelty of this study. Through mathematical analysis, it was shown that the optimal amount of added starch is 5%. This is supported primarily by the determined kinetic and thermodynamic parameters and the fact that the system did not gel during heating.

## 1. Introduction

Lithium-ion batteries are the most popular galvanic cells because of their high energy density, small weight, high voltage (app. 3.7 V), high resistance to overcharge and fast charge and discharge. Unfortunately, their main disadvantages continue to be their high cost and the flammability of their electrolytes. The second one has become a very dangerous obstacle because they have become increasingly acceptable as the main power source in electric vehicles due to their longer calendar or cycle life and increased reliability. Highly significant safety aspects include the hazards of battery vent gas (BVG) or smoke and difficulties involved in firefighting, which is caused by failures of on-board large-capacity batteries. When the temperature raises, the process is called thermal runaway and becomes a major failure mode. As obtained BVG are mainly the products of decomposition of solid–electrolyte interface (SEI) or positive electrodes materials, this failure could also be a result of different reactions between decomposition products within anode or cathode materials. For example, the BVG could be carbon dioxide, carbon monoxide, hydrogen, hydrocarbons such as methane, ethane and ethylene, and they could lead even to explosions of the lithium-ion cell [1]. Venting in an inert atmosphere may have an impact on the composed gaseous species, since it is possible that chemical species form that are unstable in mundane atmospheres [2].

The current efficiency of a lithium-ion cell decreases as the temperature drops. Temperatures above the optimum are not recommended for safety reasons, especially in a situation where the battery consists of hundreds of cells and their cooling is limited and unequal. At temperatures slightly above the limit for a given type of cell, for example, significant deposition of metallic lithium occurs. If the temperature rises significantly above the limit, the passive layer separating the negative electrode and the electrolyte may be broken. This leads to a strongly exothermic reaction of the anode, e.g., graphite, with the electrolyte. This effect can lead to pressure build-up and cell swelling. If there is free oxygen in the cell, e.g., from the decomposition of the cathode matrix caused by heat, the cell may ignite and explode. Therefore, the choice of electrolyte influences the safe operation of the cell. The electrolyte should be of good ionic conductivity (>1 mS cm^−1^), high lithium cation transfer number (>0.3), high thermal stability (−20 up to 150 °C) and high electrochemical stability (1.9–4.4 V vs. Li/Li^+^), as well as being compatible with other elements of the cell (in terms of temperatures and potentials in the cell), non-toxic and affordably priced.

Moreover, it has to be added that there exist many different failure tests described in the literature: cycled/overcharged/overdischarged, thermal-abuse (ARC, Tawerson calorimeter—Poland, Warsaw) and overdischarged (Glove Box, explosion proof room—Malsch, Germany). For tests that involved open flames or cone calorimetry, it was surmised that failure occurred in the air [3]. Flame retardants are compounds that prevent the cell from self-ignition or delay the process of thermal escape. Many requirements are imposed on them, including electrochemical inertness, physical and chemical stability, appropriate viscosity, conductivity, solubility, non-toxicity and cheapness, in addition to having an appropriate boiling point. The disadvantage is the deterioration of electrochemical properties caused by the increasing of the resistance in the circuit [4].

**Figure 1:**
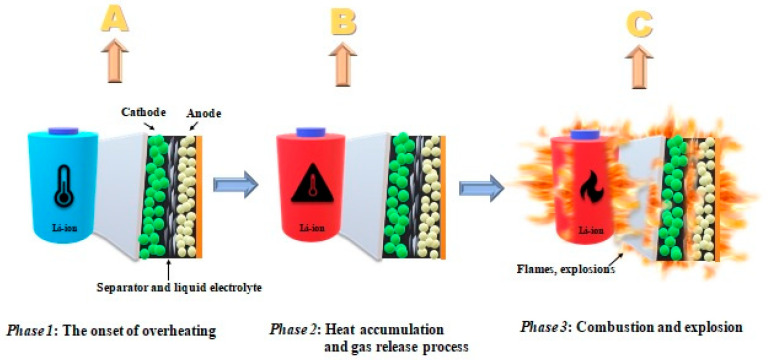
Three stages for the thermal runaway process.

The three main stages of thermal runaway, based on [5], are as follows: the onset of overheating; the heat-accumulation and gas-release process; and combustion and explosion (see Figure 1). The first stage (A) is responsible for the so-called thermal leakage of the battery. This can happen as a result of overcharging of the battery, a lack of temperature limitation or hidden cell defects. Among them, an internal short circuit is the main cause of thermal instability and is difficult to prevent. During the first stage, the battery changes its operating mode, which contributes to a significant increase in temperature. When there is a significant jump, we move from stage A to stage B.

When the temperature rises significantly in the next step (B-2), various reactions can be observed:

(a) Decomposition of solid electrolyte (SEI)—components may decompose in exothermic conditions >90 °C—for thermally unstable components;
(b) In the subsequent reaction, during the decomposition of the SEI layer, the release of highly flammable hydrocarbon gases takes place;
(c) Above >~130 °C, the separator (which melts and leads to a short circuit) is destroyed;
(d) At the very end, the cathode decomposes.

In the last step (C-3), the cell starts to burn. The electrolytes include organic compounds (cyclic and linear alkyl carbonates), which are flammable and present a hazard. In the second stage, oxygen and heat are released at the very end, which creates ideal conditions for the splashing of the organic electrolytes (causing an explosion).

Polysaccharides are a group of natural raw materials, and their application enables the increased biodegradability of the electrochemical system with appropriate energy conversion. Commonly used natural polysaccharides include cellulose, chitin, and chitosan. These biomaterials have found application in organic dye removal, heavy metal removal, and oil and solvent spillage cleanup [6]. Here, we propose the use of starch as a flame-retardant additive. Starch is a natural polymer produced by plants. As with other biopolymers (such as cellulose, chitin and glycogen), it is a low-cost, environmentally friendly and abundant material [7]. This biopolymer is exclusively composed of D-glucose residues with α-(1 → 4) linkages in linear amylose and α-(1 → 4) linkages and circa 5% α-(1 → 6) branch linkages in amylopectin [8,9,10,11,12,13,14,15,16].

## 2. Materials and Methods

### 2.1. Matreials

To the electrolyte 1 M LiPF_6_ (Lithium hexafluorophosphate) in EC:DMC (Ethyl carbonate: Dimethyl carbonate) (*v*/*v* 1:1), CS (corn starch) was added at different concentrations (5%, 10%, 20%). Additionally, the samples without starch (0%) were examined. All samples were prepared in Glove Box. All the materials came from Sigma-Aldrich (Berlin, Germany). In Table 1, all chemical compounds used in experimental part are collected.

### 2.2. Viscosity

**Figure 2:**
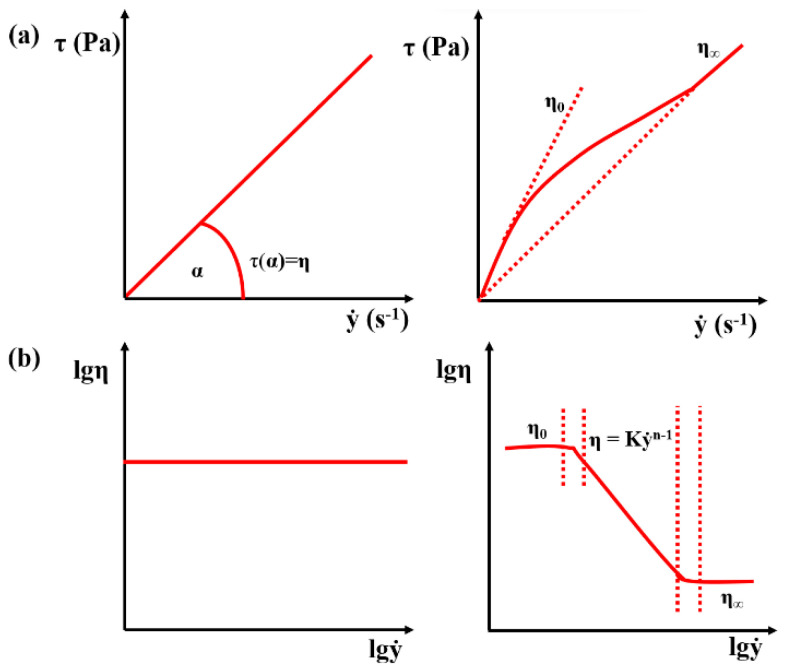
(**a**) Flow and (**b**) viscosity curves for Newtonian and non-Newtonian (shear-thinning) fluids.

A Brookfield (Viscometer model DV-I+, Brookfield Engineering Laboratories Inc., Boston, MA, USA) rotational-type viscometer was used to measure the viscosity of the pure electrolyte in EC:DMC at 25, 95 and 110 °C. Next, the same conditions were applied to examine the behavior of starch-based electrolytes (Figure 2).

In Figure 2, the viscosity and flow curves for Newtonian and reostable shear-thinning fluids are shown. Reostable fluids are fluids of which the properties are independent of time and whose flow curves are curves that begin at the point of origin. The viscosity of Newtonian fluids is independent of the shear rate. The shear-thickening and shear-thinning fluids show a power relationship—they are not satisfied by Newton’s law. To describe the behavior of those fluids, the Carreu, Ellis and Ostwald–de Waele models are commonly used.

#### 2.2.1. Self-Extinguishing Time (SET) Method

To examine the flammability of the electrolyte, the self-extinguishing time (SET) method was used. This time can be defined as follows:

(1) $$SET=\frac{t}{m}$$

In Equation (1), *t* denotes the time required for the combustion of electrolyte from ignition to extinguishment, and m is the mass of the electrolyte used in the experiment. The unit is (s g^−1^). The electrolyte is more stable when the SET parameter is smaller. There are three ranges: 6 s g^−1^ (nonflammable electrolyte), 6 s g^−1^ to 20 s g^−1^ (less flammable electrolyte) and higher than 20 s g^−1^ (flammable electrolyte) [10]. The SET value was tested on a sample at room temperature. The substance also had a flame approach, but no adscititious heating was applied. A quantity of 2 mL of the electrolyte solution was placed in the pan, and then a flame was brought to the surface of the liquid. The quantification as based on the measurement of the burning time of the sample.

#### 2.2.2. Flash Point (FP) Method

The flash point is defined as the lowest temperature at which a liquid generates flammable vapors that can be ignited in the air by a flame above its surface. The most common are the Abel and Pensky–Martens closed-cup methods (standard norms, because it is not a physico-chemical parameter) [11]. In this research, the open-cup method was utilized.

**Scheme 1:**
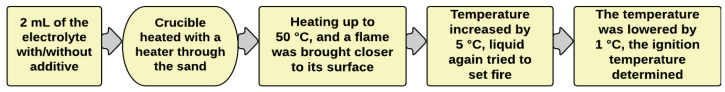
Determination of flash point step by step.

The ignition temperature test, which utilizes the Cleveland method, involves bringing the flame closer to the surface of the tested liquid with the simultaneous quantification of the solution temperature. The lowest temperature, at which the flame located above the sample will not extinguish by itself, is sought. The tests were carried out for electrolytes with sundry concentrations of the flame retardant additive. A quantity of 2 mL of the tested electrolyte was placed in a crucible heated with a heater through the sand. A thermocouple connected to a temperature gauge was immersed in the solution (Scheme 1).

In the experiment, 1 M LiPF_6_ in EC:DMC (1:1 *v*/*v*) was used. The mass of 2 mL of electrolyte was equal to 2.5595 g, while for 5%, 10% and 20% of FR (flame retardant), the equivalent mass was 0.1279, 0.2556 and 0.5119 g, respectively.

#### 2.2.3. Conductivity Measurements in Two-Electrode System

**Figure 3:**
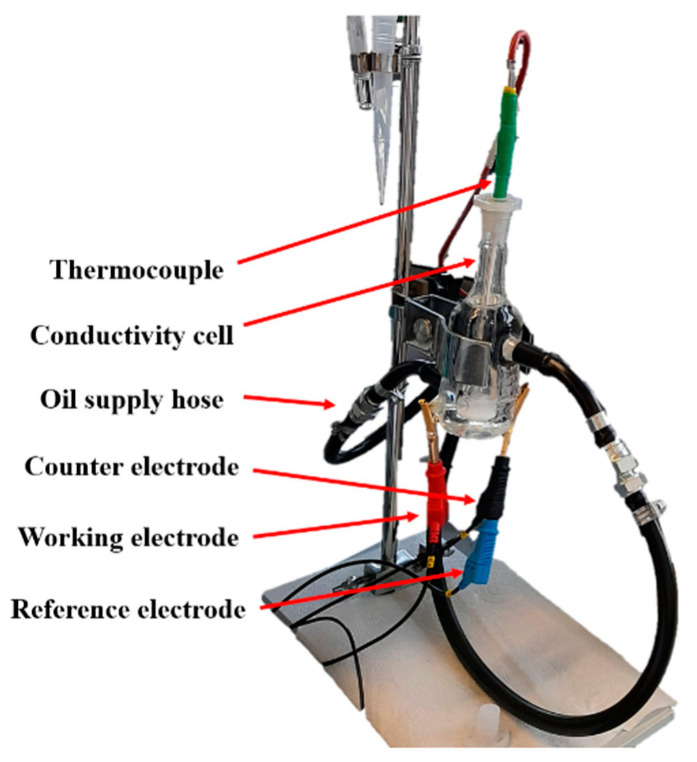
Device for conductivity measurement.

Conductivity tests at various temperatures were performed using a two-electrode system in a conductivity cell based on EIS. Using the equivalent circuit in the scheme R_el_(C_dl_(R_ct_W_Li+_)), the resistances were calculated. The determined constant k of the conductivity cell was 1.19 cm^−1^. In Figure 3, there is a device used for the measurement. Conductivity measurement for starch-based electrolyte was carried out at temperatures of 25, 50, 65, 75, 85, 95 and 100 °C, while for the pure electrolyte, the temperature was not higher than 85 °C because of the flammable properties of this compound above this temperature. Conductivity measurements of liquid electrolytes were performed in a glass cell (with a thermostating jacket) with Pt working electrodes. EIS curves were recorded with the μAutolab electrochemical system (EcoChemie, Amsterdam, The Netherlands).

#### 2.2.4. Electrochemical Examination

Among the electrochemical measurements, EIS was performed for a specific temperature range using CS, which showed the most favorable flame-retardant properties compared to other botanical origins measured (wheat starch, potato starch, rice starch). The anode was made of graphite (active material), PVdF (polyinylidene fluoride), NMP (N-Methyl-2-pyrrolidone) and acetylene black (AB). An argon atmosphere was used to prepare the electrolyte. The paste was applied to a current collector (copper plate), which was dried in an oven for 24 h at the temperature of 120 °C. The 1 M LiPF_6_ was used as the electrolyte in the EC:DMC (1:1 *v*/*v*).

The counter electrode was lithium foil (Whatmann, 0.4–0.6 mm thick). In addition, a separator was used. The entire research was carried out in an electrochemical coupling system of Swagelok^®^ type (Torun, Poland). The measurement was carried out with the use of pure electrolyte and with the addition of a flame retardant in the temperature range of 25–85 °C for pure electrolyte and up to 100 °C for electrolyte with flame retardant. The temperature was evenly distributed throughout the device. The device was equipped with double, independent systems protecting against uncontrolled temperature increases.

## 3. Results and Discussion

### 3.1. Viscosity Test

**Figure 4:**
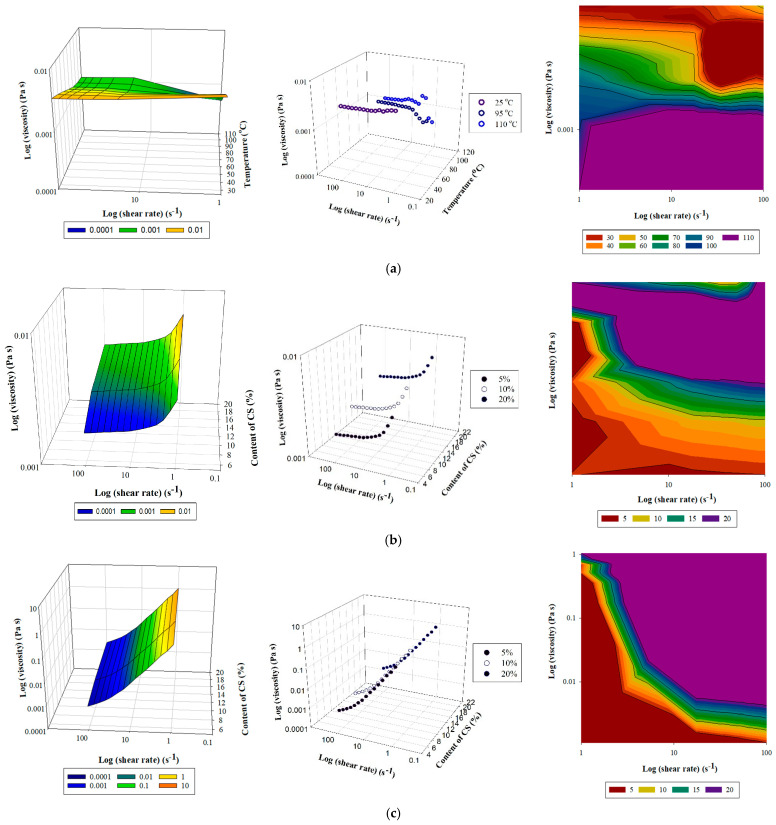
(**a**) Viscosity curves of pure electrolyte; (**b**) viscosity curves of electrolyte with CS additive at 25 °C; (**c**) viscosity curves of electrolyte with CS additive at 95°.

Pure electrolyte in the EC:DMC is a Newtonian fluid at all temperatures (25 °C, 95 °C, and 110 °C). A similar curve shape was obtained in [17]. Electrolyte with EC:DMC and starch (as a flame retardant) at 95 °C and 110 °C shows non-Newtonian properties. It was estimated that the fluid gels at 85 °C. It can be seen that the starch-based electrolyte is a shear-thinning fluid above 25 °C (Figure 4). In the case of polymer fluids, first of all, attention should be paid to their physical stability. By mentioning this, we mean the evaporation of the solvent when sealing rings or a solvent with a high boiling point is used, changes in the degree of aggregation due to the wrong dissolution procedure (repeated rheological measurements of the same solution) being used, or mechanical degradation of the chain. The rheological properties of polymeric fluids strongly depend on the molecular weight. The viscosity curves indicate that as the mean molecular weight decreases (which decreases as the temperature is increased), the zero viscosity decreases, while as the molecular weight distribution increases, the shear rate range over which the viscosity remains constant. Thus, all the dispersions studied exhibited a non-Newtonian, shear thinning flow with a tendency towards yield stress [12].

Due to the density of the pure electrolyte in solvents, amounting to 1.30 g cm^−3^ at 20 °C, it can be concluded that it is comparable to the density of glycerin. The addition of starch will increase the flow resistance and, thus, the viscosity of the sample. The shear stress of starch paste can be expressed as a function of shear rate by fitting them into different models, such as the Ostwald–de Weale, Herschel–Bulkley and Bingham models [13].

Viscosity is a physicochemical property that influences both the processes of transport and mixing of fluids as well as the transfer of heat and mass. Moreover, it is the main parameter describing the dynamic properties of fluids. Viscosity is defined as the internal friction of a fluid, i.e., it consists of a stress that depends on the strain rate. The work performed in this process takes place at the expense of the kinetic energy of the fluid in motion, which is partially transformed into the kinetic energy of the disordered movement of particles. In the case of shear deformations, the parallel layers of fluid shift against each other, accompanied by the occurrence of shear (tangential) stress. These properties are important when applied to electrolytes.

Considering the molecular theory, viscosity is the momentum exchange between parallel, contiguous layers of fluid by molecules moving from one layer to another. Molecules passing from the slower-moving layer to the faster-moving layer are the reason for the reduction in the momentum of the faster layer, and vice versa. Higher temperature increases the exchange of molecules, and thus, increases the viscosity of the gas. In a fluid, cohesive forces oppose each other’s movement. The movement of molecules is possible due to the movement of elements from one layer to another under the influence of an increase in temperature, which results in a decrease in viscosity.

#### 3.1.1. Mathematical Model of the Kinetic and Thermodynamic Determination of Fluid Flow

To determinate the Energy activation of flow *E_n_,* Equation (2) was applied [14].

(2) $$\eta =\eta_{\infty }\exp(\frac{E_{n}}{RT})$$

Then, Equation (2) was logarithmized and a graph of the dependence ln(*η*) = f (*T*^−1^) was made to obtain the *E_n_* value.

(3) $$\ln(\eta )=\ln(\eta_{\infty })+(\frac{E_{n}}{RT})$$

To determinate the Gibbs free enthalpy of decomposition, the general Eyring–Polanyi Equation (4) was used.

(4) $$k=\frac{k_{b}\cdot T}{h}\exp(-\frac{\Delta G}{RT})$$

where *k_b_* is the Boltzmann constant equal to approximately 1.3806 × 10^−23^ J K^−1^, *h* is the Planck constant equal circa 6.6261 × 10^−34^ J s and *R* is the gas constant equal to 8.314 J mol^−1^ K^−1^.

Further, to estimate the enthalpy, the combination of Equations (2) and (4) was used, given by Formula (5).

(5) E=ΔH+RT

Finally, to determine entropy of degradation, the Gibbs function (6) was used.

(6) ΔG=ΔH−TΔS

Furthermore, it was possible to calculate equilibrium constant *K* using the isotherm of van Hoff, described as follows:

(7) ΔG=−RTlnK

For homogenous dimensions, the Arrhenius activation temperature (T*) was calculated according to the work [15]. Based on the methods given in [16], the Arrhenius temperature *T_A_* was obtained.

#### 3.1.2. Kinetic and Thermodynamic Parameters According to Viscosity Test

The current research on electrolyte solutions focuses on three main areas: functional additives, fire-resistant or non-flammable electrolyte solutions, and new salts. Functional electrolyte additives are included in the solution to improve the cell performance. The technique is quite common and well developed. There are a number of additives to electrolytes, but the most important are: vinylidene carbonate, phenylcyclohexane and fluoroethylene carbonate. The selection of additives and the determination of their appropriate parameters has become an important aspect protected by patents. One of the most developed areas of research is electrolyte salts. The most commonly used salt in the electrolytes of lithium-ion cells is LiPF_6_. This salt is thermally unstable and decomposes to form LiF and PF_5_. These products react with trace amounts of water to form HF according to the following reactions:

LiPF_6_ → LiF + PF_5_

LiPF_6_ + H_2_O → POF_3_ + LiF + 2HF

PF_5_ + H_2_O → POF_3_ + 2HF

On the other hand, the reaction environment causes the cathode to dissolve and its components to enter the electrolyte. Recent research to replace LiPF_6_ is facing performance and cost issues. However, the use of a different electrolyte salt may improve the performance of the cell. An example would be LiBF_2_SO_4_ salt in EC/DMC. This cell has several advantages, including stability of cyclic operation, low cell impedance and low polarization resistance. The addition of 0.5% TFP (ethyl 3,3,3-trifluoropropane) to the classical electrolyte also improves the stability of the cyclic cathode operation at higher temperatures. It causes the formation of a stable and thin SEI layer on the electrode surface and a reduction in the interface resistance. Interesting salt proposals also include: LiBF_4_, LiTFSA, LiBETA, LiBOB and LiDFOB. These relationships have recently been widely researched.

Using the given mathematical models with the use of ionic conductivity at a given temperature, the thermodynamics and kinetics of the process were determined (Table 2).

Based on the viscosity studies, it can be concluded that at each temperature, a pure electrolyte in the mixture of organic solvents behaves in a similar manner to a Newtonian fluid. This analysis enabled the determination of specific kinetic and thermodynamic parameters of fluid flow under the influence of temperature. The low activation energy value of the flow suggests that there was a low activation barrier, which was also confirmed by the low dynamic viscosity. A positive value of free enthalpy suggests a forced nature of the reaction, while a positive value of enthalpy at constant pressure suggests an endothermic nature of the flow. The enthalpy value was relatively low, which means it required less energy for flow to occur at a given temperature. If starch was involved, its addition increased the activation energy of the process and it behaved comparably to a shear-thinning fluid in the electrolyte, which proves that it can be a great flame retardant additive for classic electrolytes.

Indeed, it can be seen that increasing the flame retardant additive increased the overall flow resistance, while the dynamic viscosity value remained close to the pure electrolyte. The value of the free enthalpy did not change significantly, which means that from the thermodynamic point of view, the flow was not significantly disturbed by reducing its spontaneity. Moreover, the entropy also remained almost unchanged, which confirms the conclusion. Thus, increasing the concentration of the flame retardant is advantageous from a thermodynamic point of view, while even a small addition significantly affects the activation energy of the fluid flow.

Moreover, the Eyring and Arrhenius plots are ideal experimental tools for describing the relationship between reaction rates and temperature, as well as for obtaining thermodynamic parameters (entropy and enthalpy), e.g., for (bio)-chemical reactions or electrolyte analysis [15,16,17,18].

Electrolyte solutions are systems that, due to their nature, cannot be described as ideal solutions, which is a significant problem for theorists dealing with the description of their thermodynamic properties. As a result of dissociation, the dissolved substance creates ions with opposite charges that interact with each other. As the electrolyte concentration increases, the importance of ion–ion interactions becomes more and more important. The electrostatic field of ions has a long range nature, although force electrostatic interactions decrease with the square of the distance between the centers of the ions. The effect of the electrostatic field is the formation of ion clouds with the opposite charge. The properties of these clouds change with temperature and distance from the central ion. The advantage of the ion–ion interaction over the ion–dipole interaction is especially visible when we consider the electrolyte with ions with valences greater than one and with high concentrations. The properties of real electrolyte solutions are determined by excess functions and activity coefficients. Determining the function of excess solutions—free enthalpy—makes it possible to determine other properties of solutions by differentiating the excess function.

The laws of chemical thermodynamics allow us to predict only the possible direction of changes in the reactant system and the number of individual components in the system when equilibrium is achieved. Given the current state of knowledge, we can learn about how the composition of the reactants in a system undergoing a chemical change changes over time, mainly empirically. The branch of physical chemistry dealing with the study of the speed of chemical reactions, determining their mechanism and the influence of various factors (e.g., the concentration of reactants, temperature, catalysts and radiation) on their course is called chemical kinetics. The results of the research on the rate of changes in the reacting system and the influence of various factors on it are often used in practice to design technologies to obtain various types of chemical products. In turn, understanding the reaction mechanism at the molecular level allows the prediction of theoretically favorable conditions for the rapid course of the designed reactions. All these parameters are important to thoroughly investigate the system that is to be used, for example, in LIBs. However, in the macroscopic set of molecules, at any moment, some of them have kinetic energy (E_k_) greater than—and others less than—the value of the average kinetic energy. This energy difference between molecules is the result of the randomness of collisions. A particle that accidentally collides with other particles in a geometrically advantageous manner can increase its speed well above the average value. Collisions between molecules are generally elastic collisions. The particles bounce off each other in a comparable manner to billiard balls, and their combined kinetic energy and momentum value remain constant. Such collisions of molecules are called inactive or ineffective, since they do not lead to the occurrence of an elementary act. From a molecular point of view, this means that the vibrational energy resources of each molecule have not changed. The average amount of kinetic energy is, therefore, too small compared to the energy quantum that could change the oscillatory state of the entire molecule. However, if they do collide coincidentally, for particles having exceptionally high E_k_ values, as a result of such a very vigorous collision, a significant increase in the amount of vibrational energy of at least one of the molecules may occur, at the expense of the total E_k_ resources of both colliding particles. This increase in the E_osc stock_ is equivalent to a weakening of the bond between the atoms that make up the molecule and, due to the close contact of E_osc-rich_ molecules, electron rearrangement may occur to form other molecules. Such a collision is called active because its consequence is the occurrence of an elementary act.

The basic kinetic and thermodynamic parameters were determined. The enthalpy of lithium ion transfer from the electrode to the electrolyte solution and the enthalpy interaction parameters were assessed. In conjunction with the Gibbs energy interaction parameters, entropy interaction parameters were also obtained. The results show that the interactions of the polysaccharides with the lithium salt may depend on the stereochemistry of the polysaccharide molecules. These interaction parameters can identify the stereochemical structure of polysaccharide molecules.

### 3.2. Flammability Tests

It is proposed that starch is used as a flame retardant due to its non-Newtonian properties. It is a shear thinning fluid (pseudoplastic fluid). Its rheological nature ensures the safety of the cell. Thus, it prevents the formation of lithium dendrites. The advantages of such a solution include the easy availability of the material, increased biodegradability of the cell, low material cost, compliance with the slogan of green chemistry, and a positive impact on the environment. In [19], the flame retardancy of wood fiber materials with phosphorus-based wheat starch was examined. Starch modified from phosphate/urea-systems is becoming a serious alternative to traditional flame retardants. In [20], the authors proposed a mixture of starch and amorphous sodium polyborate as the FR for polymers such as polyethylene terephthalate (PET) or polypropylene (PP). It turns out that the FR coating promotes starch carbonization, and the resulting layer insulates the interior against heat and oxygen, which favor ignition. On the other hand, the literature [21] mentions a separator based on a cellulose composite in LIB. Its application is aimed at reducing flammability and increasing the resistance to heat release. The separator also meets the required level of mechanical resistance, as well as demonstrating adequate absorption of electrolyte into the pores and better ionic conductivity compared to PP. A major advantage is also the increase in cyclic stability, in addition to better compatibility as well as faster transport of lithium ions. Determining the flammability parameters in a standardized way leads to a correlation between three values: ignition temperature, flame propagation speed and ignition propagation time [22,23,24,25,26,27,28,29,30,31,32,33,34,35,36].

The thermal stability of the cell is limited by the thermal stability of the electrolyte. The electrodes, current collectors and the housing have a wide temperature range in their applications. Batteries in ordinary portable devices operate at room or ambient temperature, i.e., from approx. −20 to 50 °C for most inhabited climate zones. The cell’s lower limit of useful life is determined by the freezing point of the liquid electrolyte, and the upper limit of useful life is determined by the saturated vapor pressure of the solvent, which cannot cause leakage of the casing. The upper limit is also limited by the irreversible destruction of the passive layer at elevated temperature. Exposing the cell to elevated temperatures may lead to thermal decomposition of the substances used in its construction. It is essential that the temperature at which decomposition takes place is as high as possible and that the decomposition products are harmless, which is important in extreme situations, such as fire. The use of functional additives for electrolytes is also intended to increase the temperature range of electrolyte stability in both directions. The improvement of the thermal stability of the cell is achieved not only by lowering the freezing point or reducing the vapor pressure, but also causing an upward shift in the temperature limit, in which the passive layers are irreversibly damaged.

#### 3.2.1. SET Test

**Figure 5:**
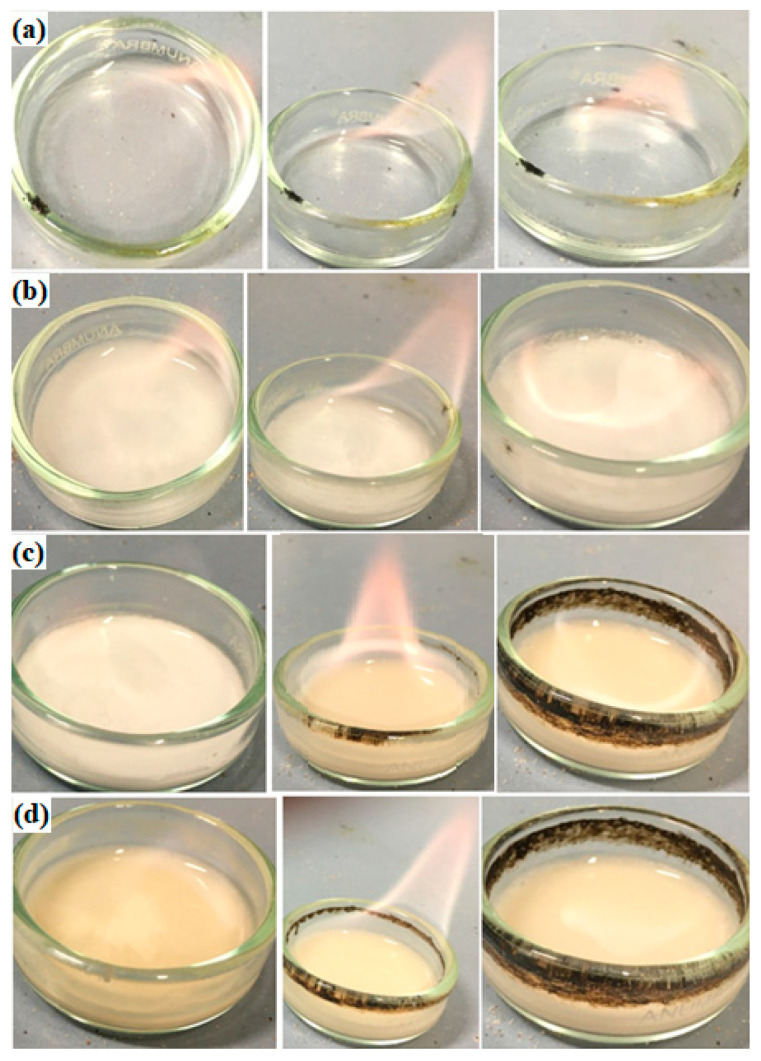
SET test using CS at the flame-retardant: (**a**) pure electrolyte; (**b**) 5% CS; (**c**) 10% CS; (**d**) 20% CS.

For each sample, three flame pictures were taken immediately after the fire was brought closer to the surface of the solution, after 30 and 50 s (Figure 5). Indeed, it can be concluded that a clean electrolyte, after being ignited, maintains the flame immediately and over time, which proves the high flammability of the electrolyte, or rather a mixture of organic solvents. With the starch addition used, a flame was visible, in all cases, after 30 s, and ceased at 50 s.

**Figure 6:**
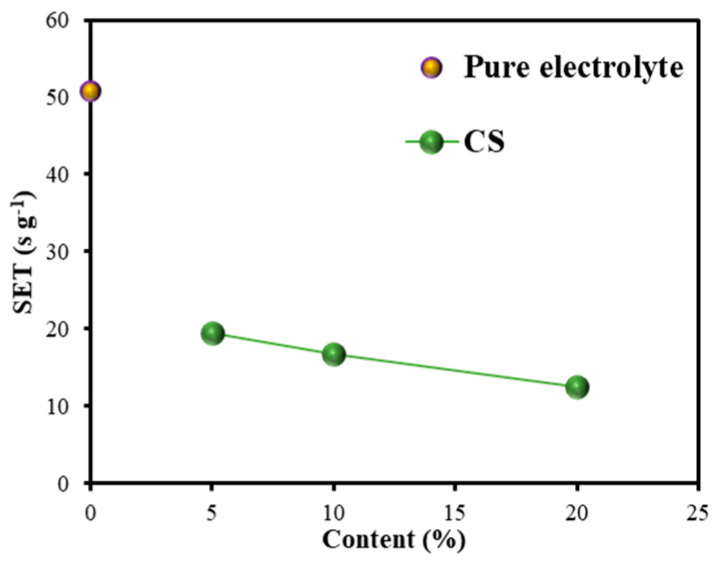
SET test using and CS and pure electrolyte.

It may be assumed that an increase in the steam sterilant content decreases the SET value (Figure 6). The addition of 10% and 20% starch produced a SET value of less than 20 s g^−1^, suggesting that the electrolyte is less flammable, while the use of a 5% additive demonstrated sufficient flame retardance to maintain the high conductivity of the electrolyte, which in pure form, has a SET point of 51 s g^−1^.

In order for a substance to be considered a suitable flame retardant, it must meet several conditions: small amounts should significantly reduce the flammability; there should be good dispersion of the additive in the electrolyte, viscosity comparable to that of a pure electrolyte, a higher boiling point than the electrolyte, little effect caused by lowering the specific conductivity, and a wide electrochemical window; in addition, it should moisturize and soak well into the electrode material and show lower reactivity in contact with other components of the cell [37]. 

#### 3.2.2. Flash Point (FP)

**Figure 7:**
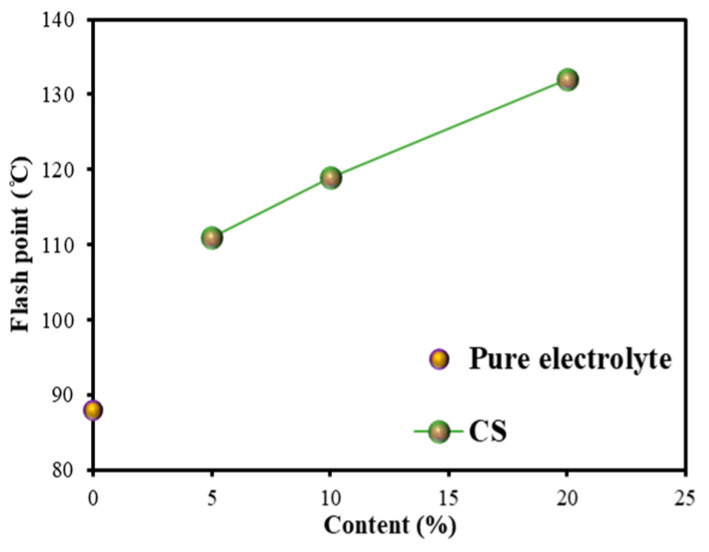
Flash point in function of content of FR.

The higher the flame retardant content, the higher the ignition temperature. It can therefore be concluded that starch significantly reduces flammability by retarding auto-ignition temperatures. The auto-ignition temperature for pure electrolyte was found to be 88 °C, while the addition of starch allowed it to be increased even to 132 °C with 20% additive being used (Figure 7). Hazardous decomposition products are formed under fire conditions. These could be carbon oxides, oxides of phosphorus, hydrogen fluoride or lithium oxides, and from the starch, these could be dimer or oligomer structures [38]. Through the decomposition of starch in this solution, some maltose structures could have been created because of the change in the color (disaccharide).

### 3.3. Conductivity Measurement

**Figure 8:**
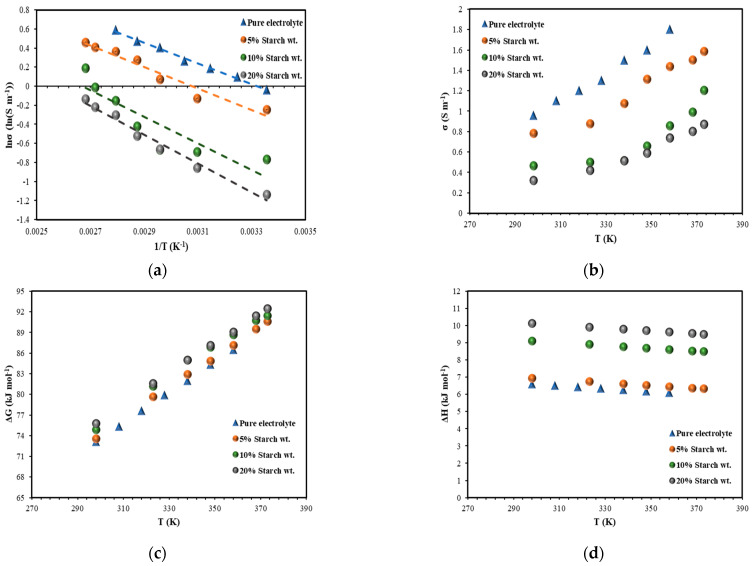
(**a**) Arrhenius equation of 1 M LiPF_6_ in EC:DMC (1/1 *v*/*v*) and with CS additive with 5%, 10% and 20% wt. additive; (**b**) dependence of specific conductivity on temperature; (**c**) dependence of Gibbs free energy on temperature; (**d**) dependence of enthalpy on temperature.

Using the relationship given by Equation (8), the specific conductivity of the electrolyte was calculated. Pure electrolyte measurements could not exceed the flash point of electrolyte in organic solvents (Figure 8).

(8) $$\kappa =k\cdot \frac{1}{R}$$

In order to determine thermodynamics and kinetics, Equations (2) and (3) were transformed into (9) and (10). The remaining equations were applied in the same way as in the case of the viscosity tests.

To determinate the Energy activation *E^#^*, Equation (9) was applied.

(9) $$\sigma =\sigma_{0}\exp(\frac{-E^{\#}}{RT})$$

Then, Equation (9) was logarithmized and a graph of the dependence ln(*σ*) = f (*T*^−1^) was made to obtain the *E^#^* value.

(10) $$\ln\sigma =\ln\sigma_{0}-\frac{E^{\#}}{RT}$$

Table 3 presents the determined thermodynamic and kinetic functions for the analyzed samples. The electrolyte was tested at temperatures ranging from 25 °C to 85 °C, and the starch electrolyte was tested in the range of 25 °C to 100 °C.

Based on the research, it can be seen that the addition of starch lowered the flash point of the pure electrolyte, which proves that it behaved as a flame retardant. With the increase in the weight content of starch in the sample, the resistance increased, which was related to a decrease in specific conductivity and, thus, an increase in the activation energy of the system. As the temperature in a pure electrolyte increases, the dissociation process takes place, and the mobility of the ions increases, which increases the conductivity of the system. It should be noted that starch slightly reduced the conductivity, as evidenced by the slightly lower activation barrier values for the initiation of active collisions. It turns out, therefore, that the addition of 5% starch was the optimal value, because the kinetics of the process were already significantly influenced with 10% starch. The positive values of the Gibbs function and the enthalpy testify to the forced nature of the process.

Solid polymer electrolytes for LIBs still suffer from poor ionic conductivity, which is lower than 10^−5^ S cm^−1^ [39]. Thus, looking at the obtained results (Figure 8), it is believed that starch could be an innovative material for the construction of the polymer electrolyte. Lithium-polymer cells are better than lithium-ion cells in terms of having higher specific capacities, and thus, increased battery life without the need to recharge. Additionally, they also provide high energy density and safety compared to commonly used liquid electrolytes [40]. The most comprehensive performance is known to be shown by composite polymer electrolytes [41]. The factors that affect the ionic conductivity are the choice of the ions, casting solvent, grain boundaries, monomer structure and stoichiometry [42]. In addition, a highly innovative idea is the use of flame-retardant polymer electrolytes as a good option for high-performance lithium metal batteries with expanded operating temperatures. The advantage of flame-retardant polymer electrolytes, besides making the batteries safer, is that their use leads to a stable SEI, good interfacial contact, low interface resistance and smooth surfaces [43,44]. The ionic conductivity influences parameters such as the maximum current density. It is an important parameter for all portable devices or electric vehicles that require a single high-power current consumption. The current density, expressed as the ratio of the current to the electrode surface, is a derivative of the internal cell resistance, which is contained in the resistance of the electrodes, electrolyte and interfacial layer. The element with the lowest conductivity (the highest resistance), which is a cathode with a conductivity of 10^−5^ S cm^−1^, limits the maximum current density. However, the conductivity of liquid electrolytes reaching 10^−3^ S cm^−1^, in addition to the resistance of the growing passive layer, also significantly affect the internal resistance of the cell; this is dependent on the salt, solvent and functional additives used.

### 3.4. Electrochemical Characteristics

Mixtures of an organic solvent and lithium salt are used as electrolytes in lithium-ion cells. The electrolyte solution must be able to freely transport lithium ions; this requires a high dielectric constant as well as a low viscosity. The physicochemical and electrochemical properties of the electrolyte depend on the nature of the organic solvent. The electrolyte must meet the following conditions: high ionic conductivity even at a relatively low temperature, low viscosity, good wettability of the separator and electrodes, high flash point, high boiling point, environmental harmlessness and low cost.

The current research on electrolyte solutions focuses on three main areas: functional additives, fire-resistant or non-flammable electrolyte solutions, and new salts. Functional electrolyte additives are included in the solution to improve the cell performance. This technique is quite common and well developed. There are a number of electrolyte additives, but the most important are: vinylidene carbonate, phenylcyclohexane and fluoroethylene carbonate.

The cell parameter values are directly or indirectly related to the properties of the electrolyte. There are several ways to improve the performance of the electrolyte: change the conductive salt, change the solvent, or optimize solvent mixture or polymer matrix. In this work, organic functional additives were used as one of the possible ways to improve the parameters of electrolytes [45,46,47,48,49,50,51].

### 3.5. EIS of the Half-Cell

Electrochemical impedance spectroscopy (EIS) is a research technique that dates back to the end of the 19th century and is related to the measurement of solid dielectric aqueous solutions, organic liquids and oxides with the Wheatstone bridge. This technique makes it possible to understand the principle of operation of electrochemical devices in which diffusion processes are tested, in addition to determining the proportion of electrolyte. The application of the technique to the study of electrochemical systems is associated with the appearance of works, in the late 1940s, that presented the concept of an electrical diagram, a circuit representing a simple electrode reaction. The development and implementation of the electrochemical spectroscopy impedance technique, has, to date, focused mainly on the use of the voltage signal as a source of excitation, where the tested system is kept under the conditions of potentiostatic control. This approach is optimal for electrochemical tests where the measurement expectations are related to impedance as a function of potential. However, there is a wide group of systems for which the use of potential control is not justified, and even causes undesirable changes in the system, so that the obtained impedance results are no longer representative. This mainly concerns corrosion systems and electrochemical energy sources in the form of batteries and cells. In addition, it is possible to obtain information on diffusion, as well as the resistance of the passivation layer or the electrolyte. Thanks to this technique, all analyzed parameters allow the study of the kinetics of the processes taking place in lithium-ion cells. This enables an analysis of the influence of temperature.

To determine diffusion coefficient *D_Li+_* (cm^2^ s^−1^), the Warburg area in the Nyquist plots was considered using Equations (11) and (12).

(11) $$D_{Li^{+}}=\frac{R^{2}T^{2}}{2A^{2}n^{4}F^{4}c^{2}\sigma^{2}}$$

where *R* refers to the gas constant (8.314 J mol^−1^ K^−1^) and *T* is a standard temperature (298 K), *A* is the effective area of the anode (1.54 cm^2^), *n* is the electronic transport ratio during oxidation, *F* is Faraday’s constant (96,500 C mol^−1^), *c* is the molar density of the Li-ion in an electrode (0.001 mol cm^−3^) and *σ* is the diffusion constant derived from the Warburg line (Ω s^−1/2^).

Furthermore, to achieve constant *σ*, the plots of *Z*′ and *Z*″ versus *ω*^−1/2^, for the range of diffusion resistances, were created, and the slope was equal to *σ* (Equation (12)).

(12) $$Z^{\prime }=R_{D}+R_{L}+\sigma \omega^{-1/2}$$

Later, the modified Arrhenius Equation (13) was applied to obtain an activation energy of the diffusion $E_{D}^{\#}$.

(13) $$\ln\frac{A_{w}}{T}=\beta -\frac{E_{D}^{\#}}{2R}\cdot \frac{1}{T}$$

To determine the kinetic parameters, it was necessary to draw a plot of ln(*A_w_* *T*^−1^) = f(*T*^−1^). The inclination angle of the obtained curve made it possible to determine the activation energy of the diffusion process ($E_{D}^{\#}$). Moreover, in Equation (13), *β* is a constant determined by the *D*_0_ (the pre-exponential factor), *A* and *c* values.

In the next step, the Nernst–Einstein law was used to determine the ionic conductivity of lithium ions (*σ_Li+_*) in the Warburg region:

(14) $$\sigma_{Li+}=\frac{cD_{Li+}n^{2}F^{2}}{RT}$$

Furthermore, to achieve the pre-expotential factor *A*, the following was proposed:

(15) $$\sigma_{Li+}=A\exp(\frac{-E_{D}^{\#}}{k_{b}T})$$

*k*_b_ is the Boltzmann constant. Then, the plot ln (*σ_Li+_*) = f(*T*^−1^) was created to obtain the intercept, which was lnA.

Subsequently, the Arrhenius equation was used to calculate the rate constant *k*:

(16) $$k=A\exp(\frac{-E_{D}^{\#}}{RT})$$

Ionic conductivity is a significant parameter in terms of assessing the efficiency of the solution used. It is used not only in electrochemistry, but also in environmental chemistry and biochemistry. It should be noted that, among other parameters, the ionic conductivity in batteries, electrodialysis devices and supercapacitors changes with the changing of the concentration and temperature, while the solvent composition usually remains the same or is not taken into account. Therefore, in order to improve the functioning of electrochemical systems, tests of electrolyte conductivity as a function of temperature and concentration should be performed. Moreover, there are already many empirical and theoretical models that show the dependence of conductivity as a function of the concentration of the electrolyte solution.

Electrolyte resistance has an influence on the results of measurements. During measurement, a drop in voltage (potential) is caused by the resistance of the electrolyte and the resistance of the charge transfer. The resistance of the electrolyte is related to the conductivity of the tested solution and depends on the amount of dissolved chemical compounds that dissociate. As a result of dissociation, ions are formed that ensure the conductivity of the electric current in the aqueous environment. The charge transfer resistance results from the properties of the electrochemical processes involved in the recombination of ions on the electrode surface. The value of this resistance is closely related to the corrosion rate, which is the basic indicator of the corrosive aggressiveness of the environment. Under the conditions of LPR measurement (direct current measurement), it is only possible to determine the polarization resistance, which is the sum of the electrolyte resistance and the charge transfer resistance: *R_P_* = *R_el_* + *R_ct_*. The resistance value of the electrolyte can be obtained from other measurements, e.g., conductivity measurement or impedance measurement.

The typical shape of a Nyquist chart consists of a semicircle and a straight line. The semicircular fragment is observed at higher frequencies; this corresponds to a process that is limited by the rate of electron transfer through the double layer. On the other hand, the linear part of the graph, which characterizes the lower frequency range, represents an electrochemical reaction controlled by the mass transfer rate, i.e., the diffusion process. In the case of reactions with fast electron transfer, the impedance spectrum may only consist of the linear part, while in the case of very slow load transfer, it consists only of the semicircle. The resulting chart shows which processes determine the characteristics of the system under study. At high frequencies, the impedance value is primarily influenced by the value of electron transfer resistance at the working electrode/solution interface. On the other hand, at low frequencies, it mainly affects the final impedance value of the Warburg impedance, which is characteristic of diffusion-controlled electrode processes. When this technique is used, the increase in the irreversibility of the reaction (slower electron transfer) causes the diameter of the observed circles to increase and the linear region of the spectrum to decrease. Full-frequency impedance measurement provides information on both types of processes. EIS is a very sensitive and relatively minimally invasive technique that allows the measurement of the electric capacity of the double layer, and of the electron transfer resistance through the double layer to the surface of the working electrode. In combination with other electrochemical techniques, it is a valuable source of information that characterizes the constructed biosensor. Impedance analysis provides information on the interface, its structure and the processes taking place there.

The obtained Nyquist curves and the calculated parameters for the proposed systems correspond to the standard ones. Therefore, one may be tempted to say that the development of starch as an anti-plating agent makes sense, and that it prompts further research on biomaterials.

**Figure 9:**
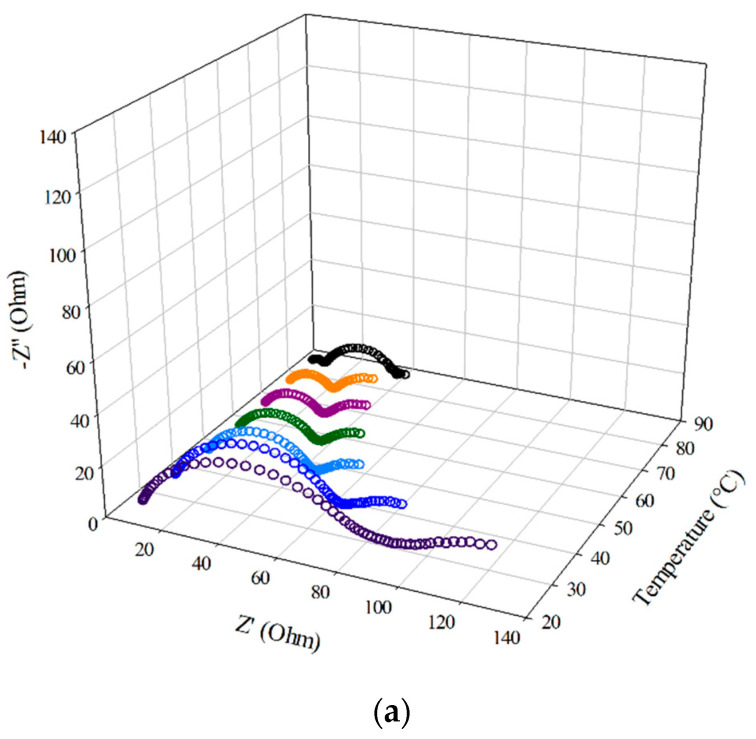

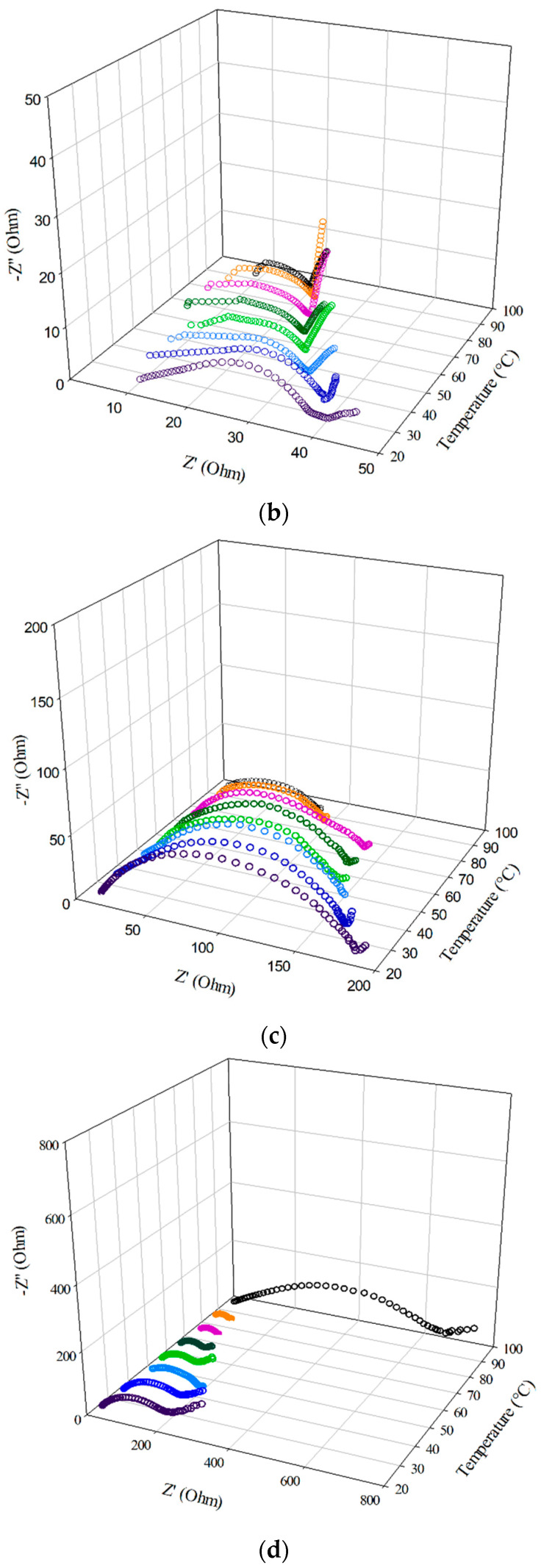

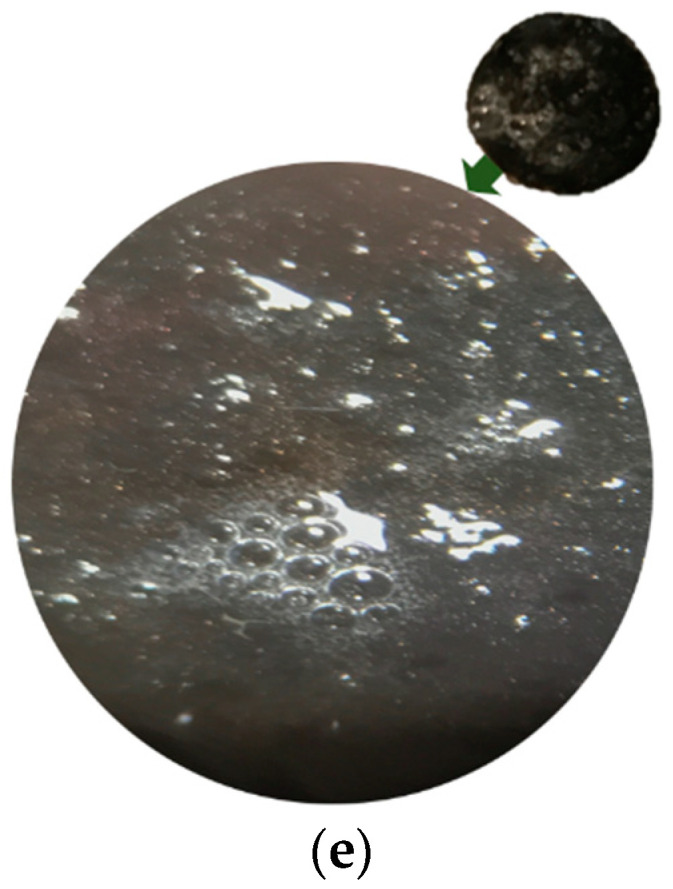
Nyquist plot of the half-cell during heating in the chamber of pure electrolyte graphite half-cell (**a**); (**b**) 1M LiPF_6_ in EC/DMC + 5% wt. starch; (**c**) Nyquist plot of the half-cell during heating in the chamber for 10% wt. starch; (**d**) 20% wt. starch (**e**) electrode image after heating (5% wt. starch).

Ion mobility is defined as the speed of an ion that moves in the direction of the field forces under the influence of an applied electric field of unit strength. The mobility of the ions increases as the temperature increases. Moreover, there is a relationship in that it is inversely proportional to the dynamic viscosity coefficient, and that it decreases with increasing concentrations of carriers. It should be noted that cations and anions have different mobilities, which is a direct result of their different structures and sizes. In addition, the ion hydration layer, upon application of an external electric field, causes the resistance to move of the ion to change during directed movement. It includes the following forces: ion–ion, solvent–solvent and ion–solvent. The increase in the concentration of the electrolyte reduces the distance between the molecules. In this case, the short-range interaction must be taken into account, which significantly increases the resistance of the moving ions. For this reason, it should be noted that a decrease in ion mobility is usually assumed when the electrolyte concentration increases. The factor that significantly influences the proper efficiency of electrochemical devices is the interfacial stability of the electrolyte with the electrode. Such stability was tested for the lithium and carbon electrodes by performing a potentiostatic test. The G | electrolyte | Li half-cell impedance response was monitored using different temperature ranges. The evolution of such a cell for an open circuit is shown as a function of temperature in Figure 9.

The cell was tested immediately after assembly without electrochemical charge/discharge. The spectra were found to consist of a flat quasi-semicircle followed by a short straight line in the low frequency region. It can be seen that the impedance dropped significantly with the temperature increased—the best effects were noticeable when 5% starch was added to the electrolyte. The electrolyte resistances were equal to 4.78, 5.31, 5.94, 8.52, 8.71, 8.86, 9.60 and 10.39 Ω for increasing temperatures (Figure 9b) and were much lower compared to the cell with higher amounts of starch (Figure 9c,d). It was also found that the interfacial resistance between the modified 5% starch electrolyte and the lithium electrode gradually increased from 5 to 12 Ω. Unfortunately, such an identical tendency was not noticed for pure electrolyte—a decrease to 75 °C and then a new increase at 85 °C was observed (Figure 9a). The electrolyte resistance values for the pure electrolyte were in the following range: 4.77, 6.26, 7.22, 7.24, 7.43, 7.97 and 8.46 Ω. It is worth taking into account the flammability and noting that the addition of starch made it possible to increase the temperature range without any significant effect on the resistivity. Considering the normal conditions, one should observe cell impedance consisting of a semicircle in the high and medium frequency range of the spectrum. These semicircles can be divided into two consecutive ones, which correspond to the SEI layer on the electrode surface (*R_SEI_*) and the charge transfer resistance (*R_ct_*) [52,53]. In this case, it is necessary to refer to gel–polymer electrolytes. Their increasing reactivity led to the formation of a thicker SEI layer and an increase in interfacial resistance. The interfacial resistance, moreover, was responsible for increasing the polarization of the cell, which directly resulted in faster loss of capacity as well as lower capacity and less cyclicity.

Moreover, after fitting of the spectrum (Figure 9d), the resistance of the electrolyte and the charge transfer were obtained. The resistance of the electrolyte was equal to 17.43, 12.40, 11.74, 11.27, 11.03, 10.96 and 15.13 Ω for increasing temperatures, while for transfer of the electrons, it was equal to 199.3, 177.5, 174.0, 111.5, 80.6, 61.1 and 733.0 Ω for increasing temperatures. Hence, it could be easily seen that for increasing temperatures, the conductivity was higher (while the resistance was lower) in the temperature range of 25–85 °C. In contrast, the resistance started to rise again at 90 °C, which was due to the gelling of the electrolyte, which was proven while measuring of the viscosity of starch-based electrolyte.

There was a tendency for 10% starch to be added to the electrolyte (Figure 9c is the same as for 5% starch (Figure 9b) only the range of resistance is different). Here, the electrolyte resistances were equal to 7.04, 9.15, 9.47, 9.91, 10.19, 10.92, 11.32 and 13.08 Ω for increasing temperatures.

Indeed, the starches that contain more amylopectin have a greater ability to change volume (granule size). Starch, in addition to the amylose and amylopectin chains, contains small amounts of ashes, proteins, fats, and also sulfur oxide (IV). It shows significant humidity, which may affect the phenomenon of swelling of the granules when the starch is left in the air. For this reason, care should be taken to keep the material in the desiccator or to dry it before use. Therefore, the phenomenon of gelling should also be taken into account; as a result of this phenomenon, under the influence of high temperature and in the presence of water, a so-called gel ball is formed. Figure 9e includes an image taken after the cooling process of the half-cell; it can be seen that, on the surface, the lithium reacted with electrode’s active materials, and thus, the oxidation process could occur.

For an electrolyte to be suitable for current and future lithium-ion cells, it should meet the following characteristics: high ionic conductivity (to minimize cell resistance and the associated heating effect of the cell, thereby reducing the risk of fire); high chemical stability (to prevent decomposition of the electrolyte on the surface of highly reactive electrode materials); high electrochemical stability (to withstand the high potential difference between the anode and cathode during cell operation); low melting point (to ensure sufficient conductivity at low temperatures and prevent solidification and phase separation); high boiling point (to ensure safety and prevent pressure build-up in the housing, which can lead to an explosion); non-toxicity and ease of recycling; and low cost (to compete with existing energy sources).

The thermal stability of the cell is limited by the thermal stability of the electrolyte. Batteries in ordinary portable devices operate at room or ambient temperature, i.e., from approx. −20 to 50 °C for most inhabited climatic zones. The lower service life limit of a cell is determined by the solidification point in the case of the liquid electrolyte, and the upper limit of the vapor pressure of the saturated solvent, which will not unseal the housing. The upper limit is also limited by the irreversible destruction of the passive layer at elevated temperature. Exposing the cell to elevated temperatures may lead to thermal decomposition of the substances used in its construction. It is essential that the temperature at which decomposition takes place is as high as possible and that the decomposition products are harmless, which is important in extreme situations, such as a fire. The use of functional additives for electrolytes is also aimed at increasing the temperature range of electrolyte stability in both directions. The improvement of the thermal stability of the cell is achieved not only by lowering the freezing point or reducing the vapor pressure, but also by shifting the temperature limit upwards, following which irreversible destruction of the passive layer occurs.

Many other anions have been developed over the past 20 years, but disadvantages of these materials were quickly discovered, preventing their use in lithium-ion cells. The most common disadvantages include the lack of sufficient ionic conductivity of the solutions of the proposed salts in solvents, which is typical for lithium-ion cells (in organic carbonates). It is assumed that such ionic conductivity, denoted by σ, should be at least 1 mS cm^−1^ at room temperature. The electrolyte can also be described as the share of lithium cations in ionic conductivity. This share describes transfer number of lithium cations. The rest of the charge is carried by the anions to which the electrodes are interlocking. Therefore, useful ionic conductivity is only found in lithium cations (*σ_Li+_*), which can be calculated from the product of the ionic conductivity and the number of lithium cation transfers. Adequate thermal stability is also required of the electrolytes in order for salt to not limit the stability of the entire cell. Organic carbonates show boiling points above 90 °C (dimethyl carbonate—91 °C); hence, they usually determine the temperature range of the cell’s operation. The salt in the solution must also be stable in terms of cell operating voltages, i.e., between 2 and 4.5 V in relation to the lithium anode. The electrochemical potential of the lithium anode is −3.04 V relative to the standard electrode hydrogen. The operating voltage limits result from the end potentials of intercalation/deintercalation with lithium cations of typical electrodes in the cell (cut-off voltage). Typical electrodes include graphite anode and cathodes: cobalt oxide, manganese oxide or nickel-manganese–cobalt mixed oxides. Using other electrodes may slightly restrict or extend the required range of stability. The stability of the salt in the solution is equally important to the other components of the cell. This is primarily relevant for materials’ electrodes, such as the mentioned oxides, but also against current collectors, including copper and aluminum. Unfortunately, most salts and solvents react with the electrode materials in terms of cell operating potentials. Thus, it is assumed that the formed electrolytes constitute stable lithium cation-permeable interfacial layers on the electrodes (abbreviated as SEI—solid–electrolyte interphase). These layers should have a low resistance values, but they cannot pass other electrolyte components, with the exception of lithium cations. SEI should also be relatively thin, i.e., they should use as few electrolyte components as possible during the increasing of the electrolyte. The interfacial layer must be stable; therefore, it cannot significantly increase or change its parameters over time. Besides, in any industry, it is important that as few toxic materials as possible are used and also that they are safe and cheap to produce. Many of the salts tested for lithium-ion cells fail in terms of the above requirements. Most are rejected at the stage of stability studies and compatibility in the presence of standard solvents and electrodes. Therefore, the most reactive materials require the highest chemical stability from the components that come into contact with metallic lithium This is why a lot of tests are carried out in its presence, although lithium in metallic form is not used in commercially available lithium-ion cells. However, using it in tests makes it possible to speed up the stability assessment of a substance or mixture.

In Table 4, the diffusion coefficient and conductivity are shown, while the whole methodology was prepared by us in a previous work [54] and can also be found in the literature [55,56,57,58,59,60,61]. The values are high, which indicates that the diffusion was efficient. Additionally, the values increased with the temperature, which is a natural process for most substances and increases heat and mass transfer [29,62,63,64,65,66].

Corn starch was proven to be a very good additive in terms of retarding the flammability of the electrolyte in a classic mixture of organic solvents. It led to both an increase in the flash point and a decrease in the SET value. The reduction in the latter flammability index indicates the increased resistance of the system to spontaneous combustion. This is essential when using lithium-ion cells in systems that require high temperatures, such as electric cars, or even for longer periods of single use of portable devices. The disadvantage of this solution is the reduction in the electrolyte conductivity and the possible occurrence of the gelling phenomenon. The advantages of using natural biomass include the increased biodegradability of the cell and compliance with the slogan of green chemistry, as well as the universal availability and easy isolation of this component.

## 4. Conclusions

Ionic conductivity is the ratio of the density of the current flowing through the body to the strength of the electric field applied to it. Its reverse is known as specific resistance. The higher the conductivity, the lower the internal resistance of the cell. In the electrolyte, the current is conducted by free ions and charged ion associations (usually triplets). The voltage forces an ion flow that is greatly limited by the interaction with electrolyte components. Conductivity therefore depends on the mobility of the ions—the higher the mobility, the higher the conductivity. Conductivity also depends on the temperature (the higher the temperature, the higher the mobility of ions, and thus the conductivity), the ion concentration, the electrolyte viscosity (the higher the viscosity, the lower the conductivity), the solvent’s electrical permeability, and the degree of salt dissociation.

Moreover, the activation energy for electrolyte—5% wt. was equal to 6.29 kJ mol^−1^, while for charge transfer, it was 21.78 kJ mol^−1^ (10% wt.–7.99 kJ mol^−1^; charge transfer: 35.22 kJ mol^−1^ and for 20% wt.–20.44 kJ mol^−1^; 53.45 kJ mol^−1^). The activation energy of the diffusion of lithium ions was placed. This value was quite low, which means there was a low activation barrier for the diffusion process; this was also proven by obtaining the rate constant k (high value). Indeed, the thermodynamic parameters indicate the presence of endothermic forces in the process of heating of the cell and its response.

Due to the non-Newtonian properties of starch, it increases work safety and prevents the production of a shear-thinned fluid in the event of a collision. The relationship between the Arrhenius viscosity parameters and the properties of the liquid at different temperatures and the content of the flame retardant may indicate how the statistical approach used here can predict the behavior and properties of other fluids, and is indicative of the universality of the model used. Moreover, it must be underlined that there should be some optimum dependence between the amount of flame retardant used and the impact on the conductivity of the electrolyte; in this study, this assigned value was 5%.

In the future, a comparison of other biopolymers to the primary electrolytes used in LIB, in terms of flammability, is planned. Cellulose and lignin will be formed between the other cells. The parameters will be extended with a larger electrochemical range.

## Figures and Tables

**Table 1:** Chemical reagents used in the experimental section.

Structure	Formula	Function
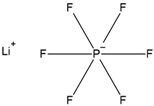	LiPF_6_	Electrolyte
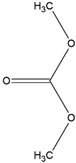	DMC	Solvent Flash point: 16 °C Boiling point: 91 °C
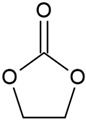	EC	Solvent Flash point: 151 °C Boiling point: 240 °C
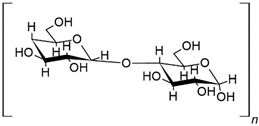	CS	Flame retardant 24.8% amylose, 0.020% phosphorus, 11% moisture [9]

**Table 2:** Average kinetic and thermodynamic parameters of pure electrolyte and with CS additive.

	Sample	1 M LiPF_6_ in EC:DMC	5% wt. CS	10% wt. CS	20% wt. CS
Parameter					
*E_η_* (kJ mol^−1^)		14.296	16.270	22.474	24.631
T* (K)		1719.51	1956.94	2703.15	2962.59
*T_A_* (K)		151.23	206.43	250.03	244.76
*η*_∞_ (Pa s)		1.15 × 10^−5^	7.65 × 10^−5^	2.02 × 10^−5^	5.54 × 10^−6^
*K* = *η* (Pa s)		1.667 × 10^−3^	1.413 × 10^−2^	2.735 × 10^−2^	1.501 × 10^−2^
Δ*G* (kJ mol^−1^)		104.710	105.982	103.921	112.651
Δ*H* = *Q_p_* (kJ mol^−1^)		11.389	13.148	19.352	21.509
Δ*S* (J mol^−1^ K^−1^)		−266.291	−247.208	−225.190	−242.691
*K* (-)		2.32 × 10^−16^	1.81 × 10^−15^	3.50 × 10^−15^	2.13 × 10^−16^

**Table 3:** Average kinetic and thermodynamic parameters of pure electrolyte and with CS additive.

	Sample	1 M LiPF_6_ in EC:DMC	5% wt. CS	10% wt. CS	20% wt. CS
Parameter					
*E^#^* (kJ mol^−1^)		9.054	9.428	11.600	12.604
*σ*_0_ = *A* (S m^−1^)		37.077	32.773	41.364	48.764
Δ*G* (kJ mol^−1^)		79.797	84.048	85.554	86.089
Δ*H* = *Q_p_* (kJ mol^−1^)		6.327	6.570	8.742	9.746
Δ*S* (J mol^−1^ K^−1^)		−223.964	−225.372	−223.437	−222.068
*K* (-)		1.96 × 10^−13^	1.69 × 10^−13^	1.02 × 10^−13^	8.4 × 10^−14^

**Table 4:** Lithium ion diffusion coefficient, conductivity *σ_Li+_* estimation of the anode at 25, 35, 45, 55, 65, 75, 85 and 90 °C (5%, 10% and 20% wt. starch) and average kinetic and thermodynamic parameters obtained for an anode with starch-based electrolyte.

T (°C)	5%	10%	20%
	*D_Li+_* × 10^−10^ (cm^2^ s^−1^)	*D_Li+_* × 10^−11^ (cm^2^ s^−1^)	*D_Li+_* × 10^−11^ (cm^2^ s^−1^)
25	3.06	4.11	8.04
35	4.37	5.22	9.43
45	6.54	7.42	13.67
55	10.5	8.33	20.11
65	18.6	9.67	32.98
75	39.6	20.11	43.45
85	56.4	34.55	52.34
90	72.3	60.23	65.89
Kinetic and thermodynamic parameters			
$E_{D}^{\#}$ (kJ mol^−1^)	44.74	49.22	68.34
*A* (S m^−1^)	2,167,316	2,248,537	2,342,174
*k* (S m^−1^)	2.4 × 10^−1^	2.9 × 10^−1^	1.8 × 10^−1^
Δ*H* (kJ mol^−1^)	42.01	58.02	60.56
Δ*G* (kJ mol^−1^)	85.55	94.34	105.65
Δ*S* (J mol^−1^ K^−1^)	−132.70	−140.32	−150.98
*K* (-)	3.33 × 10^−14^	8.52 × 10^−14^	4.21 × 10^−15^

## Data Availability

The data are available on request.
